# Supplementary material for: NASA GeneLab RNA-seq consensus pipeline: standardized processing of short-read RNA-seq data
Source: iScience. 2021 Mar 26;24(4):102361. doi: 10.1016/j.isci.2021.102361 (PMC8044432; doi:10.1016/j.isci.2021.102361)
Supplement: Document S1. Transparent methods, Figure S1, and Table S6 [file mmc1.pdf]

iScience, Volume *24*

Supplemental information

NASA GeneLab RNA-seq consensus pipeline: standardized processing of short-read RNA-seq data

Eliah G. Overbey, Amanda M. Saravia-Butler, Zhe Zhang, Komal S. Rathi, Homer Fogle, Willian A. da Silveira, Richard J. Barker, Joseph J. Bass, Afshin Beheshti, Daniel C. Berrios, Elizabeth A. Blaber, Egle Cekanaviciute, Helio A. Costa, Laurence B. Davin, Kathleen M. Fisch, Samrawit G. Gebre, Matthew Geniza, Rachel Gilbert, Simon Gilroy, Gary Hardiman, Raúl Herranz, Yared H. Kidane, Colin P.S. Kruse, Michael D. Lee, Ted Liefeld, Norman G. Lewis, J. Tyson McDonald, Robert Meller, Tejaswini Mishra, Imara Y. Perera, Shayoni Ray, Sigrid S. Reinsch, Sara Brin Rosenthal, Michael Strong, Nathaniel J. Szewczyk, Candice G.T. Tahimic, Deanne M. Taylor, Joshua P. Vandenbrink, Alicia Villacampa, Silvio Weging, Chris Wolverton, Sarah E. Wyatt, Luis Zea, Sylvain V. Costes, and Jonathan M. Galazka

Supplemental Information

Transparent Methods

The tools used in the consensus pipeline are documented in Supplemental Table 4: Pipeline Tools and Links [Table S4: “Pipeline Tools and Links, Related to Transparent Methods”]. Due to NASA security requirements, all software is updated monthly with security patching. Therefore, tool versions used to process each RNA-seq dataset hosted on the GeneLab Data Repository are provided in the RNA-seq protocol section and are also available along with exact processing scripts in the GeneLab Data Processing GitHub Repository (https://github.com/nasa/GeneLab_Data_Processing/tree/master/RNAseq/GLDS_Processing_Scripts). Specific commands, options, and flags for each tool used in the RCP are reported in the figures of the main text. Note that some packages listed here are dependencies of the packages used in the RCP. More information about such dependencies can be found in the tool documentation.

This pipeline has been run on short-read RNA-seq data in the GeneLab database (https://genelab-data.ndc.nasa.gov/genelab/projects) and is applied to new submissions to the database. Any updates to the software used in the pipeline will be noted in the Github repository GeneLab_Data_Processing (https://github.com/nasa/GeneLab_Data_Processing). There are currently over 80 RNA-seq datasets available [Table S1: “GeneLab RNA-Seq Datasets, Related to Transparent Methods”].

Processed RNAseq data from GLDS-168 and GLDS-245 select samples were used to provide an example of the downstream analyses that can be done using data processed with the consensus pipeline presented here. Normalized counts and ERCC-normalized counts from the following GLDS-168 and GLDS-245 samples were used to generate the PCA plots shown in Figure 6A & 6B and Supplemental Figure 1A & 1B, respectively. Samples from GLDS-168 and GLDS-245 that were used in this study are listed in Supplemental Table 5 [Table S5: “Sample Names, Related to Figure 6”]. Differential gene expression (DGE) data from FLT versus GC samples using (non-ERCC) normalized counts and ERCC-normalized counts data for each respective dataset were used to generate the heatmaps shown in Figure 6C & 6D and Supplemental Figure 1C & 1D, respectively. DGE data were filtered using an adjusted p value cutoff of < 0.05 and $|\log2FC|$ cutoff of > 1. The gene expression data were then sorted based on adjusted p values and the top 30 most differentially expressed and annotated genes were used to generate heatmaps with ggplot2 version 3.3.2 (Wickham, Navarro, and Pedersen 2016). Note that for visualization purposes, sample names were shortened.

Pairwise gene set enrichment analysis (GSEA) was performed on the (non-ERCC) normalized counts (Table 3) and ERCC-normalized counts [Table S6] from select samples in GLDS-168 and

GLDS-245 using the C5: Gene Ontology (GO) gene set (MSigDB v7.2) as described (Subramanian et al. 2005). All comparisons were performed using the phenotype permutation. The ranked lists of genes were defined by the signal-to-noise metric and the statistical significance were determined by 1000 permutations of the gene set. FDR <= 0.25 were considered significant for comparisons according to the authors' recommendation.

The data used to generate all PCA plots, heatmaps, and GSEA shown are provided on Mendeley (http://dx.doi.org/10.17632/fv3kd6h7k4.1).

Supplemental Figures

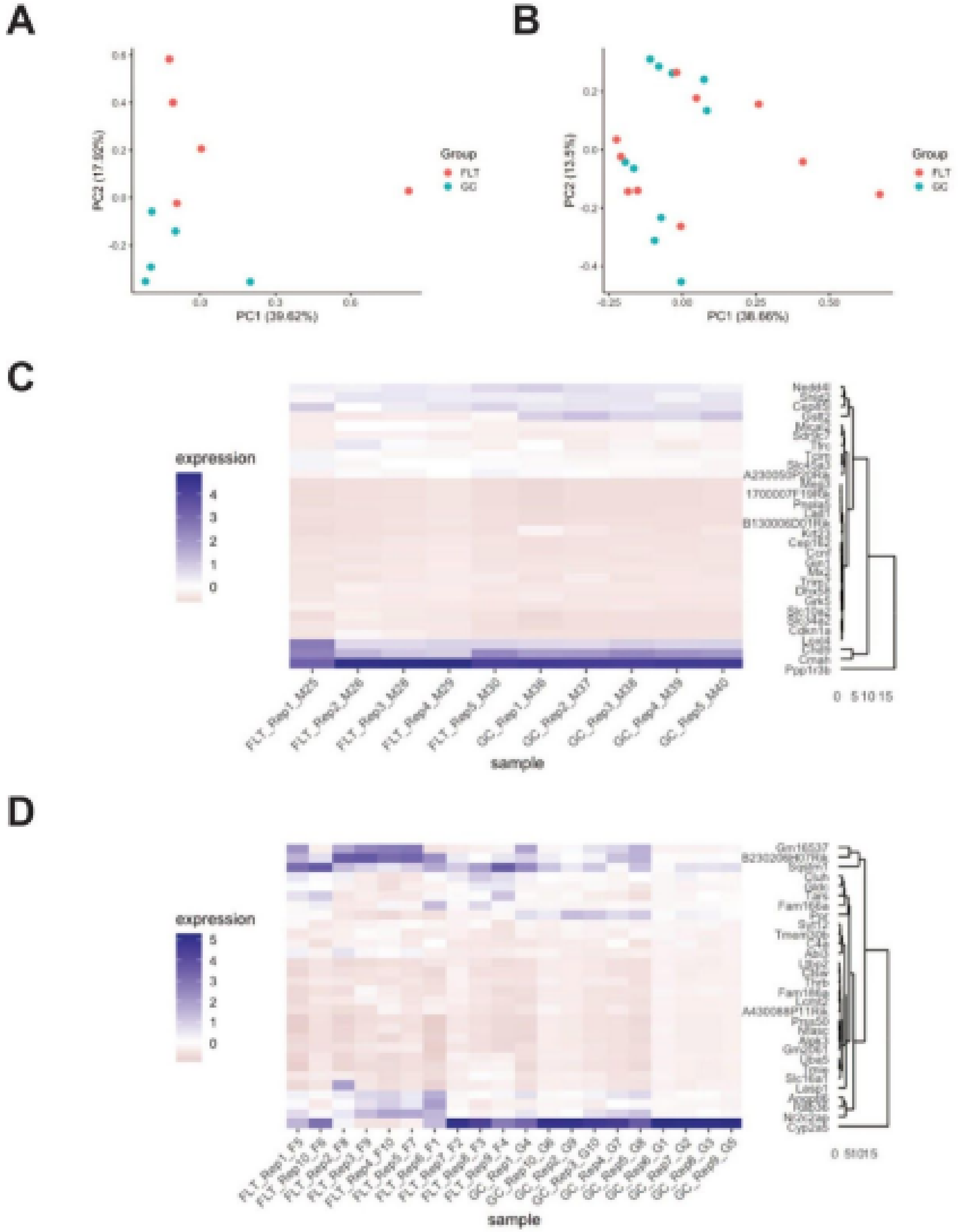
A
B
C
D
PC1 (39.62%)
PC2 (17.92%)
PC1 (36.66%)
PC2 (13.5%)
Group
FLT
GC
expression
sample
FLT_Rep1_M25
FLT_Rep2_M26
FLT_Rep3_M28
FLT_Rep4_M29
FLT_Rep5_M30
GC_Rep1_M36
GC_Rep2_M37
GC_Rep3_M38
GC_Rep4_M39
GC_Rep5_M40
Cdkn1a
Cmah
Ppp1r3b
Gm16537
Cyp2a5

Figure S1 (Related to Figure 6). Global and differential gene expression in ERCC-normalized spaceflight versus ground control liver samples from GeneLab datasets. A-B) Principal component analysis of global gene expression in spaceflight (FLT) and respective ground control (GC) liver samples from the A) Rodent Research 1 (RR-1) NASA Validation mission (GLDS-168) and B) RR-6 ISS-terminal mission (GLDS-245). Plots were generated using data in the ERCC-normalized counts tables for each respective dataset on the NASA GeneLab Data Repository. C-D) Heatmaps showing the top 30 differentially expressed genes in spaceflight (FLT) versus ground control (GC) liver samples from the C) Rodent Research 1 (RR-1) NASA Validation mission (GLDS-168) and D) RR-6 ISS-terminal mission (GLDS-245). Heatmaps were generated using data in the ERCC-normalized differential expression tables for each respective dataset on the NASA GeneLab Data Repository. Adj. p-value < 0.05 and |log2FC| > 1. All samples included were derived from frozen carcasses post-mission and utilized the ribo-depletion library preparation method.

Supplemental Tables

GeneLab Dataset	# Enriched GO terms (NOM p<0.01)	# Enriched GO terms (NOM p<0.01 & FDR<0.5)	# Enriched GO terms (NOM p<0.01 & FDR<0.25)
GLDS-168	109, 13	0, 11	0, 0
GLDS-245	166, 0	81, 0	1, 0

Table S6 (Related to Table 3). Comparison of gene ontology in ERCC-normalized spaceflight versus ground control liver samples from GeneLab datasets. The number of enriched gene ontology (GO) terms identified by Gene Set Enrichment Analysis (GSEA, phenotype permutation) was evaluated in spaceflight (FLT) versus ground control (GC) liver samples from the Rodent Research 1 (RR-1) NASA Validation mission (GLDS-168), and RR-6 ISS-terminal mission (GLDS-245). For GO terms, the number on the left corresponds to GO terms enriched in FLT samples and the number on the right corresponds to GO terms enriched in GC samples. These data were generated using the ERCC-normalized counts for each respective dataset on the NASA GeneLab Data Repository. All samples included were derived from frozen carcasses post-mission and utilized the ribo-depletion library preparation method. GLDS-168, FLT n=5 and GC n=5; GLDS-245, FLT n=10 and GC n=10. p values and FDR values are indicated.
